# KLF13 suppresses the proliferation and growth of colorectal cancer cells through transcriptionally inhibiting HMGCS1-mediated cholesterol biosynthesis

**DOI:** 10.1186/s13578-020-00440-0

**Published:** 2020-06-08

**Authors:** Weilong Yao, Yue Jiao, Yanhua Zhou, Xiaoya Luo

**Affiliations:** Department of Gastroenterology, Beijing Friendship Hospital, Capital Medical University, National Clinical Research Center for Digestive Disease, Beijing Digestive Disease Center, Beijing Key Laboratory for Precancerous Lesion of Digestive Disease, Beijing, China

**Keywords:** Colorectal cancer, KLF13, Proliferation, Cholesterol biosynthesis, HMGCS1

## Abstract

**Background:**

Colorectal cancer (CRC) is the fourth most deadly malignancy throughout the world. Extensive studies have shown that Krüppel-like factors (KLFs) play essential roles in cancer development. However, the function of KLF13 in CRC is unclear.

**Methods:**

The Cancer Genome Atlas database was applied to analyze the expression of KLF13 in CRC and normal tissues. Lentivirus system was used to overexpress and to knock down KLF13. RT-qPCR and Western blot assays were performed to detect mRNA and protein expression. CCK-8, colony formation, cell cycle analysis and EdU staining were used to assess the in vitro function of KLF13 in CRC cells. Xenografter tumor growth was used to evaluate the in vivo effect of KLF13 in CRC. Cholesterol content was measured by indicated kit. Transcription activity was analyzed by luciferase activity measurement. ChIP-qPCR assay was performed to assess the interaction of KLF13 to HMGCS1 promoter.

**Results:**

KLF13 was downregulated in CRC tissues based on the TCGA database and our RT-qPCR and Western blot results. Comparing with normal colorectal cells NCM460, the CRC cells HT-26, HCT116 and SW480 had reduced KLF13 expression. Functional experiments showed that KLF13 knockdown enhanced the proliferation and colony formation in HT-29 and HCT116 cells. Opposite results were observed in KLF13 overexpressed cells. Furthermore, KLF13 overexpression resulted in cell cycle arrest at G0/G1 phase, reduced EdU incorporation and suppressed tumor growth of HCT116 cells in nude mice. Mechanistically, KLF13 transcriptionally inhibited HMGCS1 and the cholesterol biosynthesis. Knockdown of HMGCS1 suppressed cholesterol biosynthesis and the proliferation of CRC cells with silenced KLF13. Furthermore, cholesterol biosynthesis inhibitor significantly retarded the colony growth in both cells.

**Conclusions:**

Our study reveals that KLF13 acts as a tumor suppressor in CRC through negatively regulating HMGCS1-mediated cholesterol biosynthesis.

## Background

During the past few decades, colorectal cancer (CRC) has become the fourth most lethal malignancy worldwide, with approximately 900,000 newly death annually [1]. Most of the patients receive surgical resection when diagnosed as CRC, whereas the recurrence rate is very high. Therefore, additional treatment options, such as postoperative (adjuvant) chemotherapy, preoperative (neoadjuvant) or postoperative (adjuvant) chemoradiotherapy are performed against this uncontrolled disease [2]. The well known risk factors for CRC include obesity, smoking, gut microbiota and genetic variants [3, 4]. These factors contact with each other to cause the formation of CRC.

Krüppel-like factors (KLFs), which are zinc-finger transcription factors, regulate the expression of various substrates [5]. Based on their transcription activity, KLF1–17 are divided into two different groups: (1) KLF1, 2, 4, 5, 6 and 7 act as ‘activator’; (2) the remaining functions as ‘repressor’. The role of KLFs in cancer development has been extensively investigated. KLF1, as well as KLF3 and KLF8, displays proto-oncogene in a wide range of cancers. By contrast, KLF2, KLF4 and KLF6 are more likely tumor suppressors in different cancers [6]. KLF13 gene is located at the chromosome 15. It is overexpressed in oral cancer cells. Down-regulation of KLF13 suppresses the proliferation of oral cancer cells [7]. However in prostate cancer, KLF13 functions as a tumor suppressor by inactivating AKT signaling pathway [8]. Furthermore, the FBW7-mediated degradation of KLF13 participates in the HPV life cycle and immune function [9, 10]. Nevertheless, the involvement of KLF13 in CRC is unknown.

In this study, we investigated the role of KLF13 in CRC. We found that KLF13 was downregulated in CRC tissues. Functional experiments demonstrated that KLF13 suppressed the cell cycle progression, EdU incorporation, proliferation and tumorigenesis of CRC cells. HMGCS1-mediated cholesterol synthesis was inhibited by KLF13. Furthermore, blockage of cholesterol synthesis reversed the proliferation and growth of CRC cells with lowly expressed KLF13. Our study revealed KLF13 as a tumor suppressor in CRC by regulating cholesterol metabolism.

## Materials and methods

### Patient information

Human colorectal cancer (CRC) tissues and normal tissues were collected from Beijing Friendship Hospital, Capital Medical University between 2015 and 2019. All the tissues were harvested before any therapeutic intervention. The study was carried out in accordance with the World Medical Association Declaration of Helsinki and approved by the Ethics Committee of Beijing Friendship Hospital, Capital Medical University. A written informed consent was obtained from each patient. The CRC tissues and normal tissues were subjected to RT-qPCR and Western blot analysis of KLF13.

The information of KLF13 transcript level in CRC and normal tissues was downloaded from the websites of The Cancer Genome Atlas (http://cancergenome.nih.gov). KLF13 expression was analyzed between CRC and normal tissues.

### Cell lines and cell culture

Human normal colorectal epithelial cells NCM460 and colorectal cancer cells HT-29, HCT116 and SW480 were obtained from the American Type Culture Collection. Cells were cultured 1640 medium (Hyclone), which was supplied with 10% fetal bovine serum (Gibco) and 1% penicillin/streptomycin solution (Corning). Cell culture was maintained at 37 °C with 5% CO_2_.

### KLF13 knockdown and overexpression

KLF13 was knocked down and overexpressed in HCT116 and HT-29 cells using lentivirus. For knockdown, pGCSIL-GFP, pHelper1.0 and Helper2.0 vectors were constructed and co-transfected into 293FT cells to package the lentivirus. Then the virus were filtered through 0.45 μm filters and concentrated with PEG8000. The target sequence of KLF13 was as follow: KLF13#1, CCTTACTCTGTACATAGATTT and KLF13#2, ACCAAATTGCACAATAGATAC.

For overexpression, the coding sequence of KLF13 (867 bp) was cloned into pCDH vector. The pCDH-Ctrl or pCDH-KLF13 was cotransfected with the packaging vectors PSPAX2 and PDM2G into 293FT cells. The virus were filtered through 0.45 μm filters and concentrated with PEG8000. Knockdown and overexpression efficiency was detected by RT-qPCR and Western blot assay.

### RT-qPCR assay

Total RNA from colorectal normal tissues, CRC tissues or CRC cells was isolated using the TRIzol reagent (Invitrogen), following the manufacturer’s protocols. One microgram of the RNA was reversely transcribed using M-MLV reverse transcriptase (Promega). SYBR master mixture (Takara) was used for qRT-PCR experiment on the Bio-rad real-time PCR machine. The primer sequences are listed as follow: KLF13 forward, 5′-CGGCCTCAGACAAAGGGTC-3′, and KLF13 reverse, 5′-TTCCCGTAAACTTTCTCGCAG-3′; HMGCS1 forward, 5′-CTCTTGGGATGGACGGTATGC-3′; and reverse, 5′-GCTCCAACTCCACCTGTAGG-3′; β-actin forward, 5′-CATGTACGTTGCTATCCAGGC-3′; and reverse, 5′-CTCCTTAATGTCACGCACGAT-3′; GAPDH forward, 5′-TGACTTCAACAGCGACACCCA-3′; and reverse, 5′-CACCCTGTTGCTGTAGCCAAA-3′. The expression of the indicated genes was adjusted to GAPDH in tissues or β-actin in cells.

### Western blot

Total protein was extracted from tissues or cell using RIPA buffer (Beyotime). Protein concentration was measured using BCA kit (Thermo Fisher). The proteins were separated on 10% or 12% SDS-PAGE gels, followed by transferring to PVDF membranes. Then the PVDF membranes were blocked by 5% non-fat milk and incubated with primary antibodies at 4 °C overnight. After washing with PBST, the membranes were incubated with indicated secondary antibodies for 2 h at room temperature. Protein signal was detected using the ECL-Plus kit (Amersham Biosciences). Antibody against KLF13, GAPDH and β-actin were from Proteintech. All the secondary antibodies were from Santa Cruz.

### Cell proliferation assay

CCK8 kit was used to determine the proliferation of CRC cells. Briefly, a total 3000 of shCtrl, shKLF13#1, shKLF13#2, Ctrl and KLF13 overexpressed HCT116 and HT-29 cells were seeded into 96-well plates, which contained 200 μl culture medium. Eight hours later, 20 μl CCK8 regent was added into each well and incubated at 37 °C for 3 h. Then the OD value was measured at 450 nm. The OD value at this time represents OD value at the time point of 0 h in each figure. 24, 48, 72 and 96 h later, the cell viability was determined by CCK8 kit.

### Colony formation assay

For KLF13 knockdown, shCtrl, shKLF13#1 and shKLF13#2 HCT116 and HT-29 cells were seeded into 6-well plates at the density of 1500 and 500. The cells were maintained for 8 days. For KLF13 overexpression, Ctrl and KLF13 overexpressed HCT116 and HT-29 cells were seeded into 6-well plates at the density of 2000 and 1000. The cells were cultured for 10 days. Subsequently, the colonies were formed and washed by PBS for two times. Methanol was used to fix the colonies and 0.1% crystal violet was used to stain the colonies. The colonies were photographed using the camera.

### Cell cycle analysis

Propidium iodide (PI) staining analyzed on the flow cytometer was used to detect cell cycle distribution. Briefly, the cells were seeded into the 6-well plates for three independent repeats. When reaching 80% confluence, the cells were fixed in 70% ethanol for at least 2 h. Then the cells were stained with PI and PI absorbance was detected on the C6 flow cytometer.

### EdU staining

BeyoClick™ EdU Cell Proliferation Kit with Alexa Fluor 555 was used for EdU staining, following to the manufacturer’s protocols. In brief, a total of 4 × 10^5^ HCT116 and HT-29 cells were seeded in 6-well plates, which contained coverslips. Fifteen hours later, EdU regent was added into each well and incubated at 37 °C for 4–6 h. After washing with PBS and fixing with 4% paraformaldehyde, the coverslips were incubated with 0.3% Triton X-100 and stained with Click Addictive Solution. Finally, the cells were stained DAPI.

### Cholesterol measurement

The cholesterol content was detected using the kit from the APPLYGEN company, following the manufacturer’s instructions. The cells were seed into the 6-well plates at the density of 2 × 10^6^ per well. Details were described previously. Then the OD value was measured at 550 nm [11].

### Luciferase activity measurement

The promoter of HMGCS1 was cloned into the pGL3 basic vector. HCT116 and HT-29 cells were seeded into the 24-well plates and cotransfected with indicated luciferase vectors and expression vector (knockdown or overexpression vector). Renilla luciferase vector, pCMV-RL-TK, serves as the internal control to determine the transfection efficiency.

### Chromatin immunoprecipitation (ChIP)-qPCR assay

To determine whether KLF13 binds to the promoter of HMGCS1 gene, ChIP assay was performed in Ctrl and KLF13 knockdown cells using SimpleChIP enzymatic chromatin IP kit (Cell Signaling), according to the manufacturer’s protocols. The purified DNA was subjected to qPCR analysis. The qPCR primer sequences were as follow: forward, 5′-TTTGTCCCCGCCTCTTCTC-3′ and reverse, 5′-CGATGACTCGCTAGGATTTTCC-3′. The antibodies against IgG and KLF13 were from Cell Signaling and Santa Cruz.

### Xenograft tumorigenesis assay

A total of 4 × 10^6^ Ctrl and KLF13 overexpressed HCT116 cells were subcutaneously implanted into the right forelegs of immunodeficient BALB/c nude mice (4-week-old, female). Tumor volume was calculated as V = XY^2^ (X, the longer diameter; Y, the shorter diameter). The mice were sacrificed by the day of 27 after implantation. Tumors were weighted immediately after sacrifice.

### Statistical analysis

Graphpad prism was applied to determine the statistical significance. The difference between two groups was compared by Student’s t test. One-way ANOVA was applied when more than two groups. Statistical significance was considered when p was less than 0.05.

## Results

### KLF13 is downregulated in CRC tissues

**Fig. 1:**
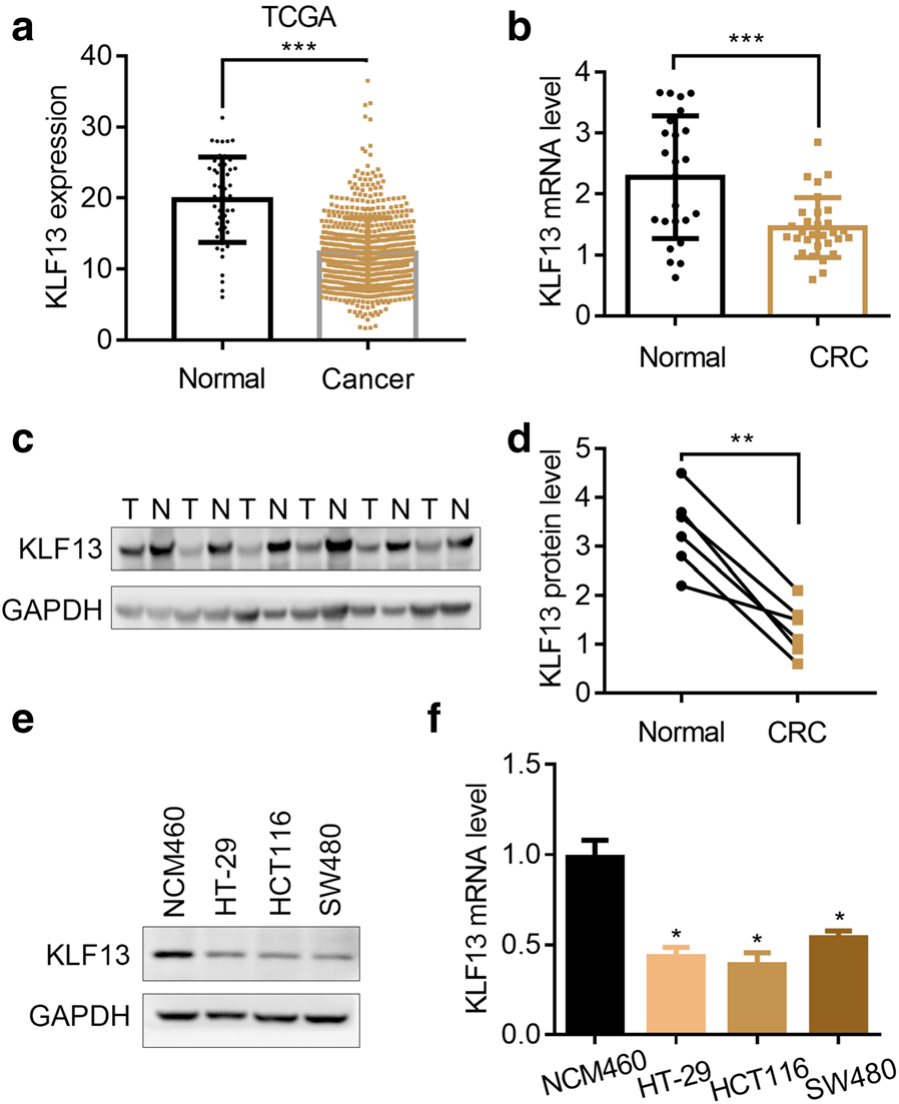
KLF13 is downregulated in CRC tissues. **a** KLF13 transcript abundance in colorectal normal tissues (n = 51) and CRC tissues (n = 647) from TCGA database. ***p < 0.001. **b** RT-qPCR analysis of KLF13 mRNA expression in colorectal normal tissues (n = 24) and RC tissues (n = 30). ***p < 0.001. **c**, **d** Western blot analysis of KLF13 protein expression in colorectal adjacent normal tissues (n = 6) and CRC tissues (n = 6). **c** Western blot images. **d** Quantification of the protein level. **p < 0.01. **e**, **f** Western blot (**e**) and RT-qPCR (**f**) analysis of KLF13 in NCM460, HT-29, HCT116 and SW480 cells. *p < 0.05

To investigate the clinical relevance of KLF13 in CRC, we collected the TCGA database and analyzed the KLF13 in CRC tissues. We found that KLF13 transcript was decreased in CRC tissues as compared with the normal tissues (Fig. 1a). To validate this result, we used RT-qPCR assay to detect KLF13 mRNA expression in CRC and normal tissues. The results showed that KLF13 abundance in CRC tissues was lower than that in normal tissues (Fig. 1b). Western blot results showed that KLF13 was downregulated in CRC comparing with its adjacent normal tissues (Fig. 1c, d). In addition, KLF13 expression was reduced in CRC cells relative to the normal cells NCM460 (Fig. 1e, f). Taken together, KLF13 is downregulated in CRC tissues and maybe correlated with disease progression.

### KLF13 suppresses the proliferation and growth of CRC cells

**Fig. 2:**
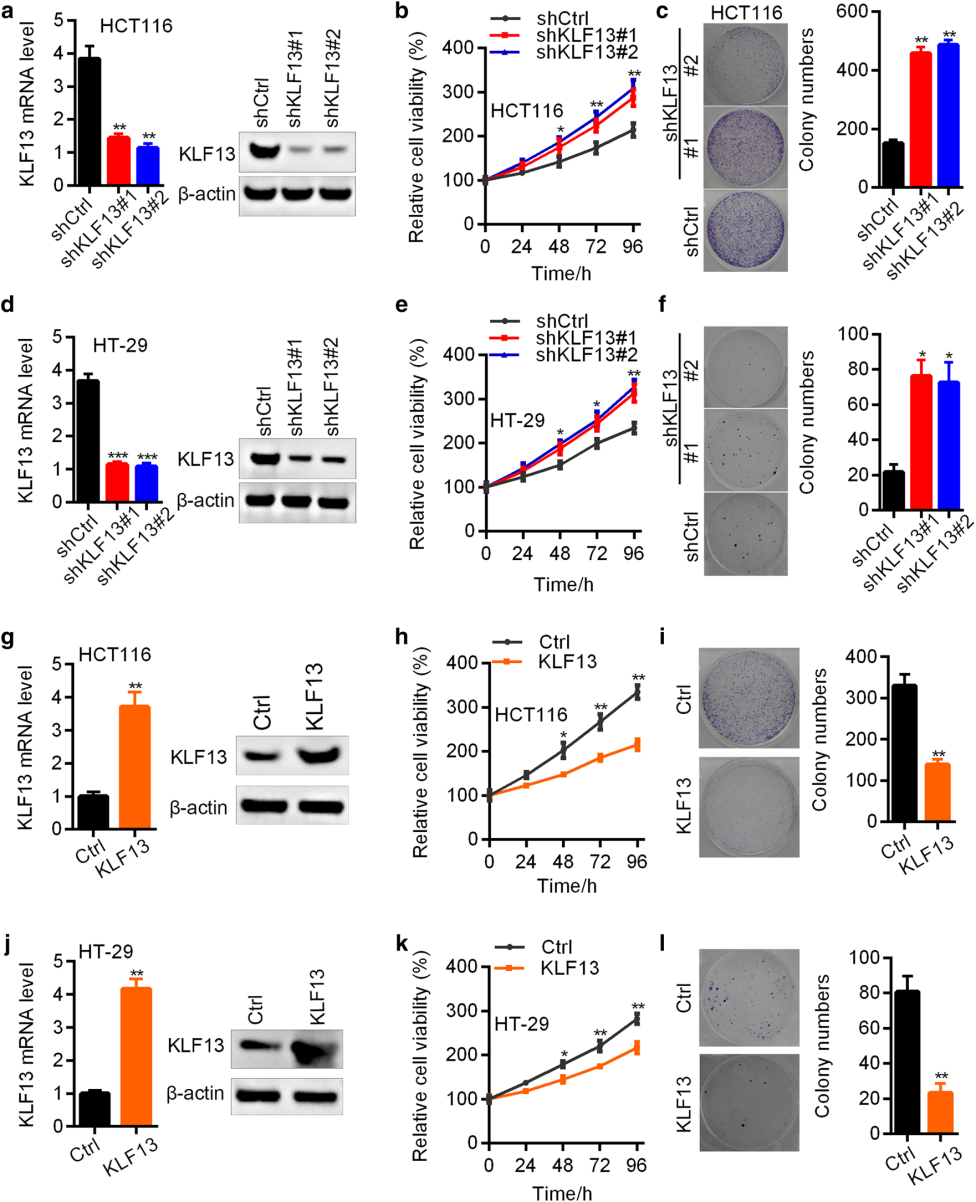
KLF13 suppresses the proliferation of CRC cells. **a**–**c** shCtrl, shKLF13#1 and shKLF13#2 HCT116 cells were subjected to RT-qPCR and Western blot analysis of KLF13 (**a**), CCK8 analysis of cell proliferation (**b**), and colony formation assay (**c** Left, representative images; Right, quantification results). *p < 0.05, **p < 0.01. **d**–**f** shCtrl, shKLF13#1 and shKLF13#2 HT-29 cells were subjected to RT-qPCR and Western blot analysis of KLF13 (**d**), CCK8 analysis of cell proliferation (**e**), and colony formation assay (**f** Left, representative images; Right, quantification results). *p < 0.05, **p < 0.01, ***p < 0.001. **g**–**i** Ctrl and KLF13 overexpressed HCT116 cells were subjected to RT-qPCR and Western blot analysis of KLF13 (**g**), CCK8 analysis of cell proliferation (**h**), and colony formation assay (**i** Left, representative images; Right, quantification results). *p < 0.05, **p < 0.01. **j**–**l** Ctrl and KLF13 overexpressed HT-29 cells were subjected to RT-qPCR and Western blot analysis of KLF13 (**j**), and CCK8 analysis of cell proliferation (**k**), and colony formation assay (**l** Left, representative images; Right, quantification results). *p < 0.05, **p < 0.01

To validate the role of KLF13 in CRC, we knocked down and overexpressed KLF13 in CRC cells HT-29 and HCT116 using lentivirus. RT-qPCR and Western blot results demonstrated that KLF13 was efficiently knocked down in HCT116 cells (Fig. 2a). Down-regulation of KLF13 promoted the proliferation and colony growth of HCT116 cells (Fig. 2b, c). Consistent results were also found in KLF13 silenced HT-29 cells (Fig. 2d–f). To confirm the function of KLF13 in CRC, we overexpressed KLF13 in both cells. KLF13 was overexpressed in HCT116 and HT-29 cells. Up-regulation of KLF13 suppressed the proliferation and colony growth of both cells (Fig. 2g–l). Collectively, KLF13 is a tumor suppressor in CRC.

### KLF13 overexpression promotes cell cycle arrest and inhibits the DNA synthesis in CRC cells

**Fig. 3:**
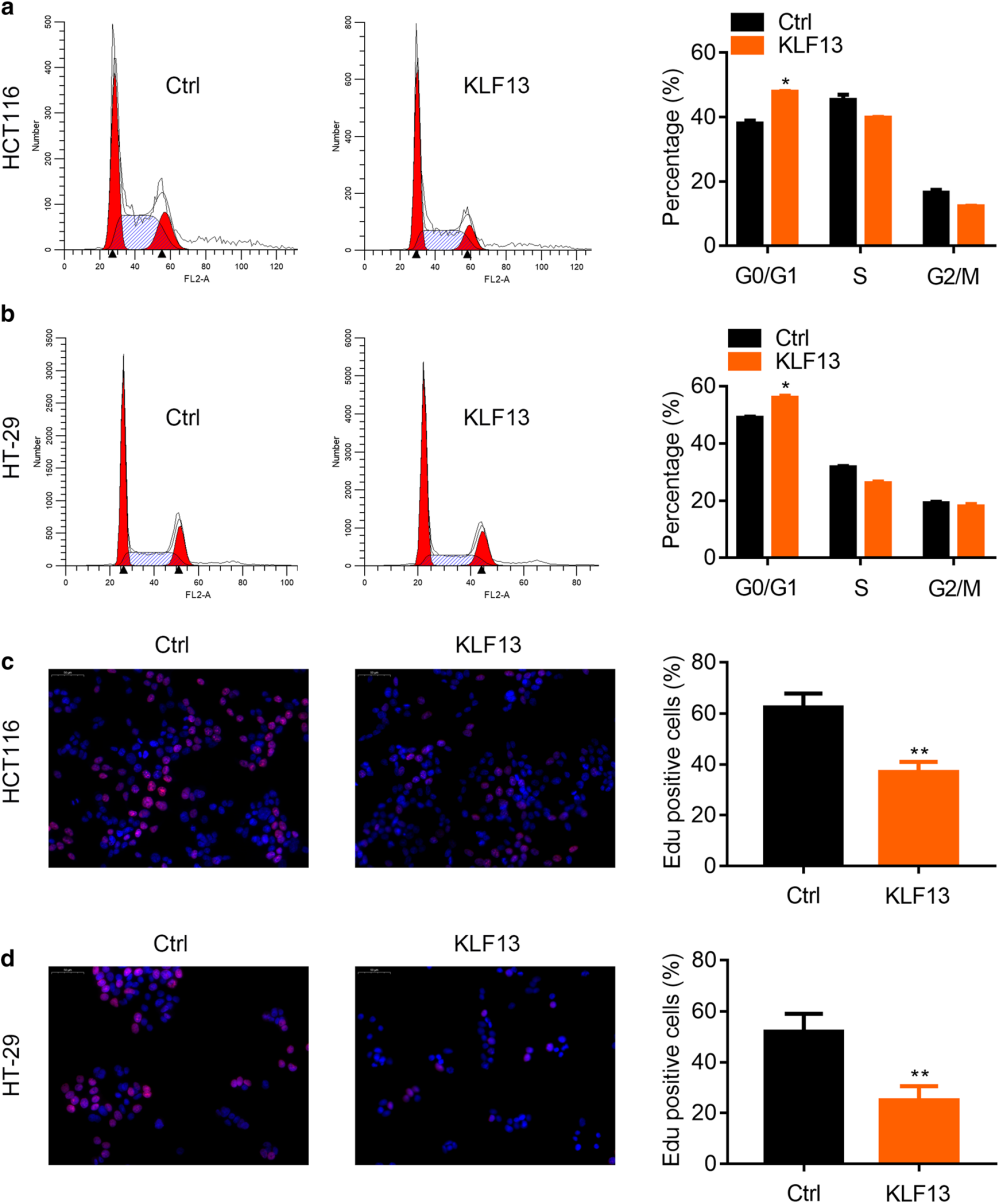
KLF13 inhibits cell cycle progression and DNA synthesis in CRC cells. **a** Ctrl and KLF13 overexpressed HCT116 cells were subjected to flow cytometry analysis of cell cycle. Left, representative images. Right, quantification results. *p < 0.05. **b** Ctrl and KLF13 overexpressed HT-29 cells were subjected to flow cytometry analysis of cell cycle. Left, representative images. Right, quantification results. *p < 0.05. **c** Ctrl and KLF13 overexpressed HCT116 cells were subjected to EdU staining. Left, representative images. Right, quantification results. **p < 0.01. **d** Ctrl and KLF13 overexpressed HT-29 cells were subjected to EdU staining. Left, representative images. Right, quantification results. **p < 0.01

Next, we analyzed the role of KLF13 in cell cycle progression and DNA synthesis. The HCT116 and HT-29 cells transfected with Ctrl or KLF13 overexpressed lentivirus were subjected PI staining and flow cytometry analysis of cell cycle. The results showed that KLF13 overexpression led to disrupted cell cycle distribution, with increased G0/G1 and decreased S and G2/M phase. This suggested that KLF13 promoted cell cycle arrest at G0/G1 phase (Fig. 3a, b). EdU incorporation is an indicator of DNA synthesis. We then analyzed whether KLF13 regulated DNA synthesis by detecting EdU staining. We found that KLF13 ectopic expression resulted in weaker staining of EdU in HCT116 and HT-29 cells (Fig. 3c, d). These results indicate that up-regulation of KLF13 suppresses the cell cycle progression and DNA synthesis.

### KLF13 overexpression suppresses the xenografted tumor development

**Fig. 4:**
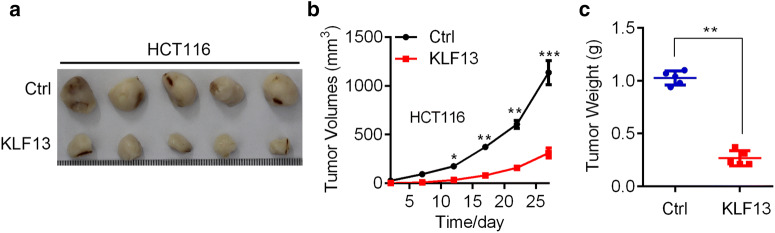
KLF13 suppresses the xenografted tumorigenesis of HCT116 cells. **a**–**c** Ctrl and KLF13 overexpressed HCT116 cells were subcutaneously implanted into the 4-week-old immunodeficient female nude mice. **a** Representative images of the tumors. **b** Tumor growth curve. **c** Tumor weight. *p < 0.05, **p < 0.01, ***p < 0.001

To explore the in vivo role of KLF13, we subcutaneously implanted the Ctrl and KL13 overexpressed HCT116 cells into the right forelegs of 4-week-old immunodeficient female nude mice. HCT116 Ctrl cells started to develop tumor by day 3 after implantation, while KLF13 overexpressed HCT116 cells formed tumors by day 7. Overall, KLF13 overexpression obviously inhibited the xenografted tumor initiation and progression of HCT116 cells in vivo (Fig. 4). Our results highlight the suppressive role of KLF13 in tumorigenesis.

### KLF13 inhibits cholesterol biosynthesis through transcriptionally suppressing HMGCS1 expression

**Fig. 5:**
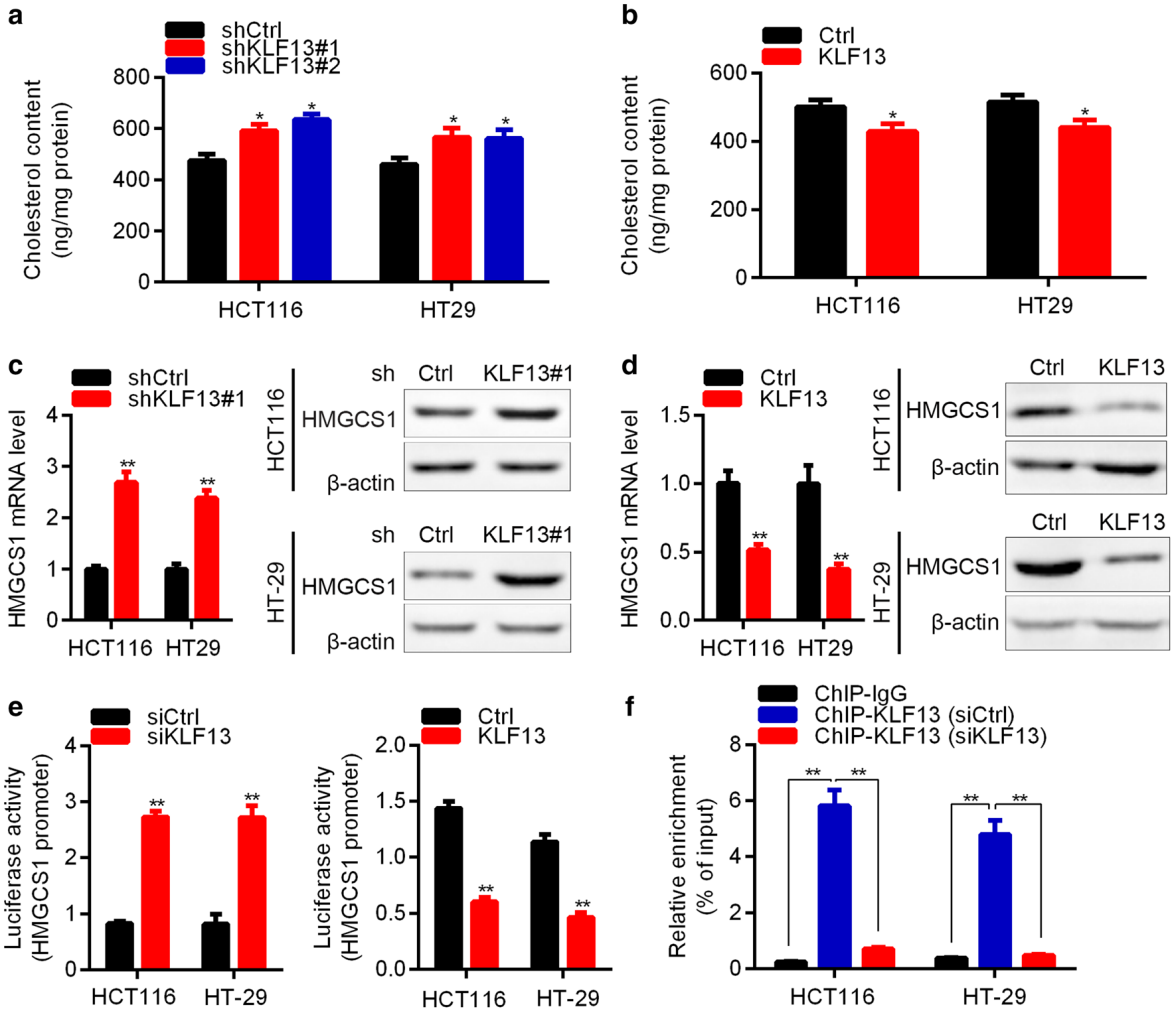
KLF13 reduces cholesterol biosynthesis and transcriptionally represses HMGCS1. **a** Cholesterol content was detected in shCtrl, shKLF13#1 and shKLF13#2 HCT116 and HT-29 cells. *p < 0.05. **b** Cholesterol content was detected in Ctrl and KLF13 overexpressed HCT116 and HT-29 cells. *p < 0.05. **c** shCtrl and shKLF13#1 HCT116 or HT-29 cells were subjected to RT-qPCR and Western blot analysis of HMGCS. **p < 0.01. **d** Ctrl and KLF13 overexpressed HCT116 or HT-29 cells were subjected to RT-qPCR and Western blot analysis of HMGCS. **p < 0.01. **e** Luciferase activity of HMGCS1 promoter was measured in siCtrl, siKLF13, Ctrl and KLF13 overexpressed HCT116 and HT-29 cells. **p < 0.01. **f** ChIP-qPCR assay was performed in KLF13 knockdown HCT116 and HT-29 cells to determine KLF13 binding to HMGCS1 promoter. **p < 0.01

Cholesterol biosynthesis benefits for cancer cell proliferation. We then analyzed whether KLF13 regulated cholesterol biosynthesis in CRC cells. Firstly, shCtrl and shKLF13 CRC cells were subjected to cholesterol content detection. The results showed that KLF13 knockdown increased the cholesterol level in both HCT116 and HT-29 cells (Fig. 5a). Likewise, KLF13 ectopic expression suppressed the cholesterol biosynthesis in CRC cells (Fig. 5b).

HMG-CoA synthase 1 (HMGCS1) is an important factor responsible for cholesterol biosynthesis. We checked whether KLF13 regulated the expression of HMGCS1. RT-qPCR and Western blot results showed that KLF13 knockdown upregulated HMGCS1 at the mRNA and protein level in HCT116 and HT-29 cells (Fig. 5c). In contrast, KLF13 overexpression resulted in down-regulation of HMGCS1 in HCT116 and HT-29 cells (Fig. 5d). To confirm whether KLF13 regulates HMGCS1 at transcriptional level or nor, we performed luciferase reporter assay. KLF13 was knocked down and overexpressed in CRC cells and the pGL3 basic vector containing HMGCS1 promoter was co-transfected into the CRC cells. We found that KLF13 knockdown in HCT116 and HT-29 enhanced the luciferase activity, whereas KLF13 overexpression suppressed the luciferase activity (Fig. 5e). Importantly, ChIP-qPCR results showed that KLF13 bound to the promoter of HMGCS1 gene (Fig. 5f).

### Knockdown of HMGCS1 and inhibition of cholesterol biosynthesis suppress the proliferation of CRC cells with silenced KLF13

**Fig. 6:**
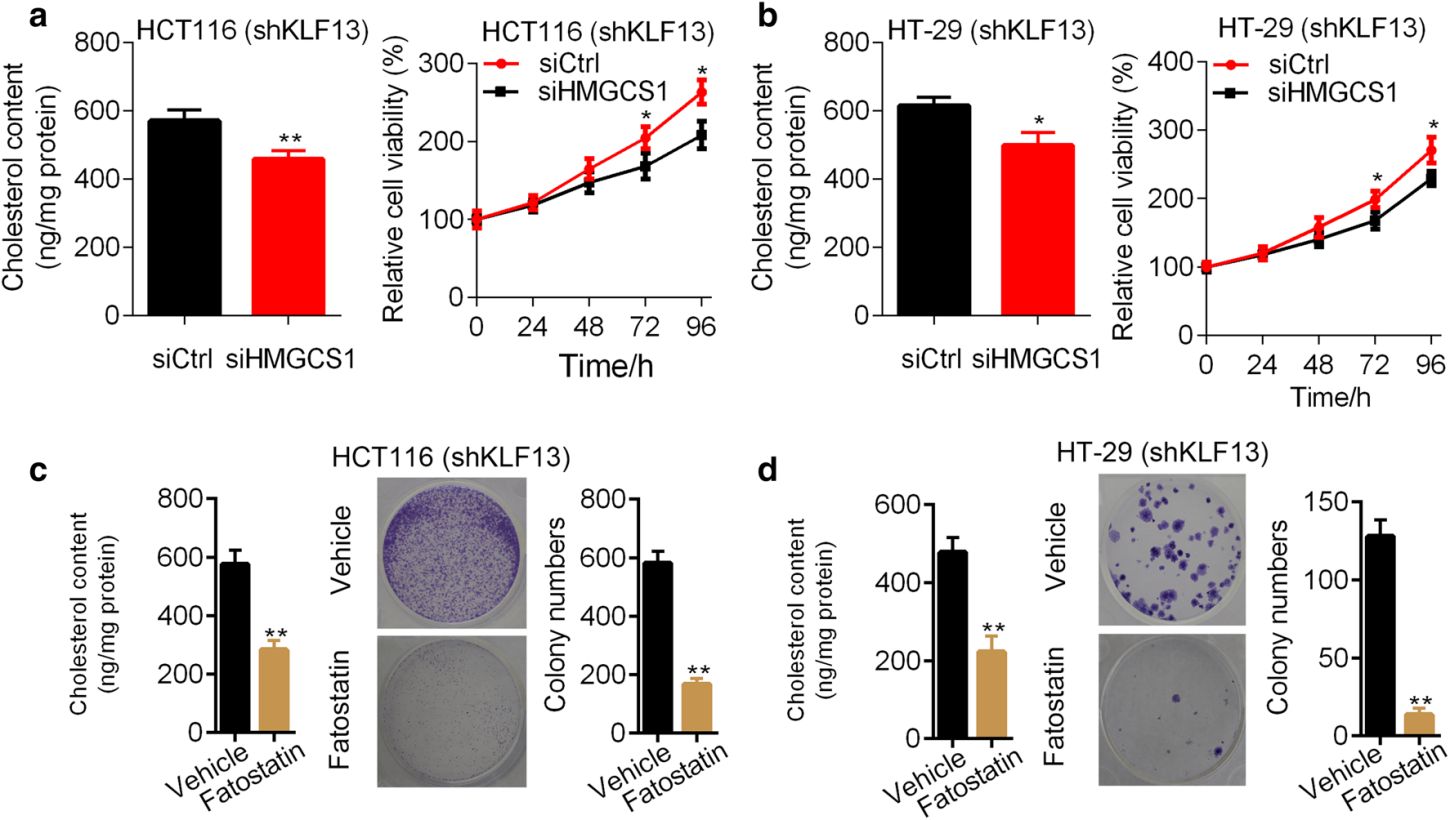
HMGCS1 and cholesterol biosynthesis blockage suppresses the proliferation and growth of CRC cells. **a** shKLF13 HCT116 cells transfected with siCtrl or siHMGCS1 were subjected to cholesterol detection and CCK8 analysis of proliferation. *p < 0.05, **p < 0.01. **b** shKLF13 HT-29 cells transfected with siCtrl or siHMGCS1 were subjected to cholesterol detection and CCK8 analysis of proliferation. *p < 0.05. **c** shKLF13 HCT116 cells were treated with or without fatostatin were subjected to cholesterol detection and colony formation assay. **p < 0.01. **d** shKLF13 HT-29 cells were treated with or without fatostatin were subjected to cholesterol detection and colony formation assay. **p < 0.01

Since cholesterol biogenesis may promote the growth of cancer cells, we knocked down HMGCS1 in KLF13 knockdown CRC cells. Downregulation of HMGCS1 decreased the cholesterol abundance in KLF13 knockdown HCT116 cells (Fig. 6a). Importantly, KLF13 silencing inhibited the proliferation of HCT116 cells with KLF13 reduction (Fig. 6a). In addition, HMGCS1 knockdown retarded the cholesterol biosynthesis and cell growth in KLF13 silenced HT-29 cells (Fig. 6b).

We next used fatostatin to treat KLF13 knockdown HCT116 cells and HT-29 cells. Fatostatin significantly reduced cholesterol contents in HCT116 and HT-29 cells (Fig. 6c, d). Colony formation was significantly suppressed by fatostatin in these cells (Fig. 6c, d). Collectively, downregulation of HMGCS1 and cholesterol biosynthesis inhibitors reverses the enhanced cell viability triggered by KLF13 silencing.

## Discussion

Increased cholesterol is positively correlated with the development of CRC [12]. However, the up-stream controller of cholesterol biosynthesis in CRC needs to be determined. In this study, we found that KLF13 negatively regulated cholesterol biogenesis in CRC cells. KLF13 was downregulated in CRC tissues. Down-regulation and up-regulation of KLF13 promoted and inhibited the proliferation of CRC cells. HMGCS1, which is critical for cholesterol synthesis, was suppressed by KLF13 at the transcriptional level. Blockage of cholesterol by either HMGCS1 interference or drugs reduced the viability of CRC cells with lowly expressed KLF13.

There are 17 members of KLFs family, including KLF1–17. Even though most of them recognize the ‘CACCC’ sequence at the promoter region of down-stream genes [13], they exhibit distinct transcriptional activity because of their ability to interact with different co-activators or repressors [14]. Therefore, KLFs display different physiological and pathological functions. Recently, KLFs are found to play important roles in cancer development, including CRC. KLF2 acts as a tumor suppressor in CRC via regulating HIF-1α/Notch-1 signaling pathway [15]. KLF4 overexpression enhances the stemness and mesenchymal features in CRC cells [16]. KLF6 is a tumor suppressor, while KLF8 functions as an oncogene in CRC [17, 18]. Nevertheless, the precise function of KLF13 in CRC remains to be determined. Here, we identified KLF13 as a tumor suppressor in CRC. KLF13 expression was reduced in CRC tissues and its silencing promoted the proliferation of CRC cells. Ectopic expression of KLF13 resulted in suppressed cell cycle progression, DNA synthesis and proliferation of CRC cells. Our study demonstrated that KLF13 expression was negatively associated with the development of CRC.

The HMG-CoA synthases (HMGCS), including HMGCS1 and HMGCS2, are important enzymes that promote the de novo synthesis of cholesterol [19, 20]. Suppression of HMGCS1 could reduce the proliferation of colon cancer cells [21]. In addition, *Peptostreptococcus anaerobius* induces the biogenesis of cholesterol and growth in colorectal cancer cells. The HMGCS1 is upregulated by *Peptostreptococcus anaerobius* and functions as an oncogene [22]. We found here that KLF13 downregulated HMGCS1. Luciferase reporter assay showed that KLF13 negatively regulated the transcriptional activity of HMGCS1. ChIP-qPCR results verified the binding of KLF13 to the promoter of HMGCS1 gene. Our study reveals the transcriptional regulation of HMGCS1 by KLF13 in CRC.

We also observed that knockdown of HMGCS1 reduced the cholesterol content and the viability of CRC cells with suppressed KLF13. These results suggest that up-regulation of HMGCS1 contributes to the tumor suppressive role of KLF13 in CRC. However, whether the increased cholesterol synthesis in KLF13 silenced CRC cells contributed to CRC cell proliferation and growth should be addressed. The results will help us gain some insights into the treatment options for CRC patients with lowly expressed KLF13. Fatostatin is a SREBP inhibitor, which significantly suppresses the synthesis of fatty acid and cholesterol. Fatostatin exhibits anti-tumor effect by blocking SREBP-regulated metabolic pathways [23]. Here, we used Fatostatin to treat KLF13 silenced CRC cells. We observed that fatostatin exhibited a higher inhibitory effect on cholesterol contents and cell proliferation than that HMGCS1 knockdown did in KLF13 silenced HCT116 and HT-29 cells. These results suggest that fatostatin maybe more effective than HMGCS1 inhibition for CRC patients with lowly expressed KLF13.

## Conclusion

In summary, we demonstrated that KLF13 served as a tumor suppressor in CRC through negatively regulating HMGCS1-mediated cholesterol biosynthesis. Down-regulation of KLF13 was observed in CRC tissues and contributed to the accelerated proliferation, growth and tumorigenesis of CRC cells. Molecular experiments showed that KLF13 transcriptionally repressed the promoter region of HMGCS1. Silencing of HMGCS1 or inhibition of cholesterol biogenesis reversed the malignant phenotypes in KLF13 silenced CRC cells. Our study highlights the role of KLF13 in CRC by regulating cholesterol metabolism.

## Data Availability

All data generated or analyzed during this study are included in this published article.
